# Long-Term Safety and Immunogenicity of a Tetravalent Live-Attenuated Dengue Vaccine and Evaluation of a Booster Dose Administered to Healthy Thai Children

**DOI:** 10.4269/ajtmh.15-0659

**Published:** 2016-06-01

**Authors:** Veerachai Watanaveeradej, Sriluck Simasathien, Mammen P. Mammen, Ananda Nisalak, Elodie Tournay, Phirangkul Kerdpanich, Rudiwilai Samakoses, Robert J. Putnak, Robert V. Gibbons, In-Kyu Yoon, Richard G. Jarman, Rafael De La Barrera, Philippe Moris, Kenneth H. Eckels, Stephen J. Thomas, Bruce L. Innis

**Affiliations:** Department of Pediatrics, Phramongkutklao Hospital, Bangkok, Thailand; Department of Virology, United States Army Medical Component–Armed Forces Research Institute of Medical Sciences (USAMC-AFRIMS), Bangkok, Thailand; GSK Vaccines, Rixensart, Belgium; Viral Diseases Branch, Walter Reed Army Institute of Research, Silver Spring, Maryland; Dengue Vaccine Initiative, International Vaccine Institute, Seoul, Republic of Korea; Pilot BioProduction Facility, Translational Medicine Branch, Walter Reed Army Institute of Research, Silver Spring, Maryland; GSK Vaccines, Philadelphia, Pennsylvania

## Abstract

We evaluated the safety and immunogenicity of two doses of a live-attenuated, tetravalent dengue virus vaccine (F17/Pre formulation) and a booster dose in a dengue endemic setting in two studies. Seven children (7- to 8-year-olds) were followed for 1 year after dose 2 and then given a booster dose (F17/Pre formulation), and followed for four more years (Child study). In the Infant study, 49 2-year-olds, vaccinated as infants, were followed for approximately 3.5 years after dose 2 and then given a booster dose (F17) and followed for one additional year. Two clinically notable events were observed, both in dengue vaccine recipients in the Infant study: 1 case of dengue approximately 2.7 years after dose 2 and 1 case of suspected dengue after booster vaccinations. The booster vaccinations had a favorable safety profile in terms of reactogenicity and adverse events reported during the 1-month follow-up periods. No vaccine-related serious adverse events were reported during the studies. Neutralizing antibodies against dengue viruses 1–4 waned during the 1–3 years before boosting, which elicited a short-lived booster response but did not provide a long-lived, multivalent antibody response in most subjects. Overall, this candidate vaccine did not elicit a durable humoral immune response.

## Introduction

Dengue, the most common global arthropod-borne viral disease, is caused by any of four dengue viruses (DENV 1–4), single-stranded RNA viruses of the genus *Flavivirus*.

The burden of dengue has increased over the past decades with an estimated annual global incidence of 100 million symptomatic cases including 1 million severe cases and 21,000 deaths.^1^,^2^ Close to 75% of the global disease burden of dengue is found in the southeast Asia and the Western Pacific regions.^3^ In 2013, there were approximately 155,000 cases of dengue reported by the Ministry of Public Health in Thailand (where all four DENV types are endemic^4^), with a case fatality rate of approximately 0.1%.^5^ The primary vector, *Aedes aegypti*, is widely distributed across Thailand and a secondary vector, *Aedes albopictus*, has expanded significantly in recent years.^5^

Development of effective tetravalent dengue vaccines is a high public health priority in endemic regions.^6^ The U.S. Army Medical Research and Materiel Command (USAMRMC) in partnership with GSK Vaccines in Belgium developed a live-attenuated, tetravalent DENV vaccine as four separate monovalent vaccines (DENV-1, -2, -3, and -4) that were mixed before administration. This candidate vaccine, DENV F17/Pre formulation (referred to as F17/Pre hereafter), was previously described.^7^ The F17/Pre vaccine was found to be well tolerated and immunogenic in a phase 2 trial involving U.S. adult volunteers.^8^ The vaccine was further tested in two phase 1/2 clinical trials in Bangkok, Thailand: one small study in healthy children 6–7 years of age^8^ and the other in healthy infants 12–15 months of age.^9^

The trial in children was an open study in which seven Thai children received two doses of the F17/Pre vaccine, 6 months apart. The children had no detectable neutralizing antibodies to any DENV serotype or to Japanese encephalitis virus (JEV) before the first vaccination. Based on the favorable safety profile observed in that first pediatric trial, a randomized, observer-blind, controlled trial was conducted in 51 infants, 12–15 months of age, who also had no detectable neutralizing antibodies to any DENV serotype or to JEV. Infants received two doses of F17/Pre vaccine 6 months apart (either a 1/10 dose, or full dose) or control vaccines.

In both studies, the F17/Pre vaccine was well tolerated with no serious adverse events (SAEs) related to the vaccine or clinically significant laboratory values following the two-dose vaccination series. One child experienced fever (38.2°C, < 2 days) and associated DENV-4 vaccine viremia 7 days after dose 2. Approximately 10 weeks after dose 2, five of the six children in the per protocol analysis exhibited neutralizing antibodies ≥ 1:10 to all four DENV serotypes (referred to as a tetravalent response).^8^ Among those who received the full-dose F17/Pre vaccine in the Infant study, 53.6% developed a tetravalent response.^9^

Subsequently, we initiated two studies in which the same Thai children and infants in Bangkok (where dengue is highly endemic) were followed up to 5 years after the two-dose primary vaccination series to assess the persistence of anti-DENV antibodies and the occurrence of hospitalization due to dengue. In addition, we evaluated whether these immunized children and infants would respond to a booster dose of live-attenuated DENV vaccine given either 12 months or 41–46 months, respectively, after the completion of the primary vaccination. These two follow-up/booster studies are herein referred to as the Child study and the Infant study.

The booster dose given in the Child study was the same F17/Pre vaccine given in the primary vaccination phase to both the children and infants. However, for the Infant study, a re-derived tetravalent version of the vaccine was given (details provided in section Vaccines of Materials and Methods). The re-derived vaccine (TDEN/F17, referred to F17 hereafter), was initially evaluated in a study of dengue-naive adults in the United States,^10^ along with the F17/Pre vaccine. That study showed that there were no major safety or immunogenicity differences between the formulations. Two subsequent studies in a dengue-endemic region (Thailand and Puerto Rico, respectively) confirmed the safety and immunogenicity of the F17 vaccine.^11^,^12^ On the basis of these positive results, the F17 vaccine was administered in the Infant study as a booster dose.

Here, we report on data collected in these two long-term follow-up/booster Child and Infant studies.

## Materials and Methods

### Study design and objectives.

**Figure 1.**
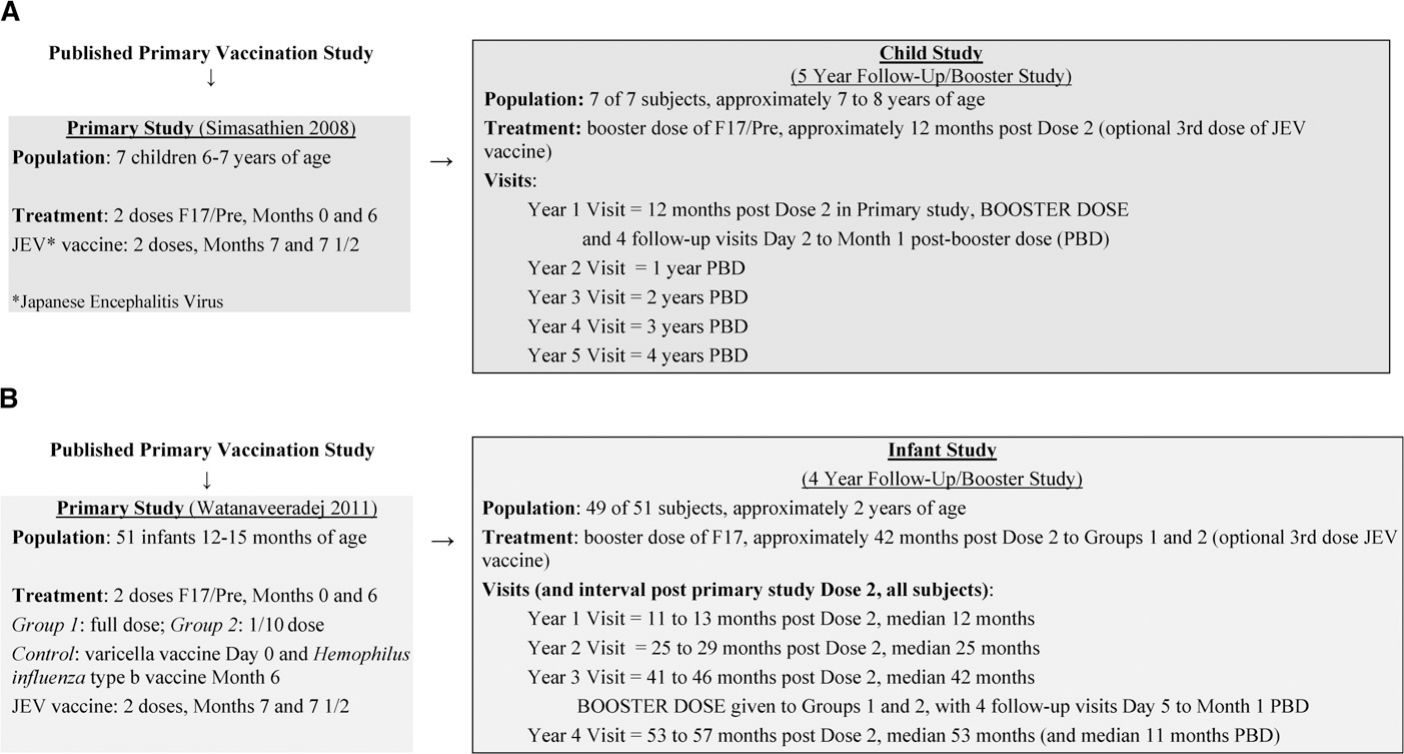
Study designs and cohorts, (**A**) Child study and (**B**) Infant study.

Both the Child and Infant studies were phase 1/2 trials designed to follow subjects for 4 and 5 years, respectively, after they had received two doses of the live-attenuated F17/Pre vaccine 6 months apart in the primary studies (Figure 1

). In both cases, the subjects were reenrolled in an open manner (i.e., at the end of the primary vaccination trials, the treatment groups were known by the parents and study personnel).

The Child and Infant studies (NCT00318916 and NCT00322049), respectively, were conducted between 2005 and 2009, in accordance with Good Clinical Practice guidelines, the provisions of the 1996 version of the Declaration of Helsinki and both the US and Thai regulations. The study documents, including the clinical protocols and informed consent forms were reviewed and approved by the ethical review committees of the Royal Thai Army, the U.S. Army Office of the Surgeon General, and Walter Reed Army Institute of Research (WRAIR).

In the Child study, the booster dose of F17/Pre vaccine was given approximately 12 months (year 1 visit) after completion of the primary vaccination, and subjects had follow-up visits at years 2, 3, 4, and 5 (post primary vaccinations) as shown in Figure 1A.

In the Infant study, subjects had follow-up visits approximately 1 and 2 years after completion of the primary vaccination and received a booster dose of F17 vaccine 41–46 months (approximately 3.5 years) after dose 2 in the primary study, at the year 3 visit. Only the infants who previously received the F17/Pre vaccine were given a booster dose of F17, although the infants from the control group were included in the follow-up for safety over the 4 years. The last study visit for infants occurred 1 year after the booster dose, at the year 4 visit (refer to Figure 1B for study visit timings).

**Figure 2.**
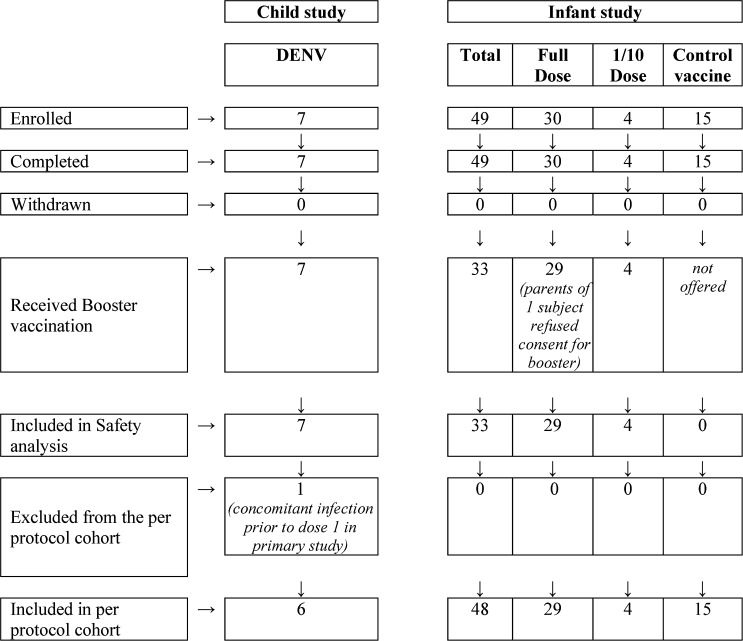
Disposition of subjects in long-term follow-up and booster studies.

The disposition of subjects in both studies is shown in Figure 2

.

The objectives of the long-term follow-up studies were to assess the levels of anti-DENV neutralizing antibodies and any abnormal safety laboratory measures 12 months (Child study) or 42 months (Infant study) after primary vaccination completion. We also assessed the occurrence of dengue, particularly illness prompting hospitalization, over either 4 or 5 years post primary vaccination. The objectives of the two studies were similar except that the booster dose was administered later in the Infant study with only 12 months of follow-up post booster dose for levels of anti-DENV neutralizing antibodies and dengue, with no safety laboratory endpoints collected beyond 30 days post booster.

### Study participants.

At the time of reenrollment into the follow-up/booster studies, which occurred approximately 6 months to 1 year after completion of the primary studies, the children were 7–8 years of age and the infants were approximately 2 years of age (Figure 1).

In the Child study, children were enrolled in the primary vaccination study from the Wat Samian Naree School in Bangkok, which was identified as a study population with historically low Japanese encephalitis incidence and vaccination rates. Subsequently, these children were invited to continue in the long-term follow-up and booster study and thus were reenrolled in 2005 (1 year after the second dose of primary vaccination). This long-term follow-up/booster study was completed in 2009. All seven children in the 5-year follow-up/booster study previously received two doses of the DENV vaccine and two doses of JEV vaccine (Figure 1A). Details of enrollment into the primary study were previously reported.^8^

All infants were recruited into the primary vaccination study from the Department of Pediatrics, Phramongkutklao (PMK) Hospital, Bangkok, and had received two doses of either the F17/Pre vaccine (full dose or 1/10) or control vaccines followed by two doses of JEV vaccine (Figure 1B).^9^

The primary vaccination study was completed in 2005 and the parents were invited to reenroll their children into the follow-up study. This long-term follow-up/booster study was completed in 2009.

The cohorts, study visits, and intervals since dose 2 in the primary vaccination study are shown in Figure 1 for each study.

### Role of sponsor and development partners.

The sponsor for both studies was USAMRMC. The studies were co-funded, designed, monitored, and reported by USAMRMC and GlaxoSmithKline Biologicals SA. Investigators from PMK Hospital and the Armed Forces Research Institute of Medical Sciences (AFRIMS) performed the study and collected and encoded the data into a GSK Vaccines database, and a GSK Vaccines statistician analyzed the data according to a prespecified and mutually approved plan.

### Vaccines.

As previously mentioned, the F17/Pre booster vaccine administered in the Child study was the same vaccine formulation administered to children and infants as their two-dose primary vaccination. Details regarding the vaccine development, preparation, and potency testing by immunofocus assay were previously reported in those studies.^8^,^9^ Vaccine potency is shown in Table 1. This F17/Pre vaccine was developed at the WRAIR and manufactured at the Salk Institute Government Services Division, Swiftwater, PA. Four monovalent freeze dried vaccines (shown in Table 1) were rehydrated and mixed in equal volumes and administered as a tetravalent DENV vaccine. A volume of 1.0 mL was injected subcutaneously in the upper triceps/deltoid area.

The booster dose given to subjects in the Infant study was the re-derived F17 vaccine previously described in the studies in the United States, Thailand, and Puerto Rico.^10^–^12^ The F17 vaccine was manufactured by the Pilot Bioproduction Facility, WRAIR, Silver Spring, MD. A volume of 0.5 mL was injected subcutaneously in the upper triceps/deltoid area as a booster dose. Viral concentrations for booster doses are provided in Table 1.

In the Child study, a dose of licensed inactivated JEV vaccine (Beijing strain, Government Pharmaceutical Organization of Thailand) was injected subcutaneously 1 month after the dengue booster vaccination. In the Infant study, the same JEV vaccine dose was offered at the nominal year 1 visit in accordance with Thailand's Expanded Program for Immunization.

#### Surveillance for dengue.

Dengue surveillance (collecting information from parents/guardians regarding all emergency room, outpatient clinic, and physician office visits for suspected dengue) was performed at enrollment in the follow-up study and then at subsequent yearly study visits, supplemented by mid-year phone contacts. Overall, there were five annual visits post-primary dose 2 in the Child study and four annual visits in the Infant study. Actual intervals of each study visit from the dose 2 visit are provided in Figure 1. For the purposes of this report, yearly visits will be referred to as the year 1 visit, year 2 visit, and so forth, although as shown in Figure 1, year 3 for the Infant study actually occurred at a median time of 3.5 years post dose 2 and year 4 occurred at 4.4 years post dose 2.

### Safety assessments following booster vaccination.

Refer to Supplemental Figures 1–4 for safety assessments following the booster vaccination.

### Immunogenicity assessments.

For DENV and JEV serology, blood specimens were collected at the year 1 visit (before booster vaccine administration), 30 days post booster vaccination, and at the year 2 and year 3 visits for the subjects in the Child study and at the year 1, year 3 (before booster vaccine administration), 30 days post booster vaccination, and year 4 visits for the subjects in the Infant study.

Neutralizing antibodies to each DENV serotype were measured at Viral Diseases Branch, WRAIR using a plaque-reduction neutralization test (PRNT_50_) as previously described.^8^,^13^ Each serum sample for DENV analysis was screened at a dilution of 1:10 against the four DENV serotypes using the vaccine parent strains (i.e., DENV-1 WP74, DENV-2 S16803, DENV-3 CH53489, and DENV-4 341750). Specimens that were positive by virtue of demonstrating a 50% or greater reduction in virus plaques were endpoint titered. Seropositivity to DENV was defined as a PRNT_50_ titer ≥ 1:10 dilution.

JEV neutralizing antibodies were measured using a PRNT_50_ assay with 4-fold dilutions of sera. The assay was performed at United States Army Medical Component–AFRIMS (Bangkok, Thailand) using the attenuated JEV SA_14_-14-2 strain. Seropositivity to JEV was defined as a titer ≥ 1:10 dilution.

Evaluation of cell-mediated immune response included frequencies of DENV-specific memory B cell measured using B-cell enzyme-linked immunospot (ELISPOT), and specific CD4^+^ T cells and CD8^+^ T cells by cytokine flow cytometry. Testing was performed at GSK Vaccines, Rixensart, Belgium, on blood samples collected at year 3 in the Child study and at three time points in the Infant study (just before booster vaccination, 30 days post booster, and 1 year post booster vaccination).

The B-cell ELISPOT allowed the quantification of antigen-specific memory B cells. The assay involved the incubation of peripheral blood mononuclear cells (PBMCs) that were differentiated into antibody secreting cells in polyvinylidene difluoride plates coated with either a recombinant truncated 80E protein subunit of the serotype being analyzed (Hawaii Biotech, Inc., Honolulu, HI) for the detection of antigen-specific memory B cells, or antihuman IgG for the detection of total memory B cells.^14^ A conventional immuno-enzymatic procedure was applied to detect antibody/antigen spots enumerating memory B cells, and the results were expressed as the frequencies of antigen-specific memory B cells within the total memory B-cell population.^14^

We used an intracellular cytokine staining (ICS) assay to quantify dengue-specific memory T cells following the booster vaccination. Exploratory T-cell response measurements included ICS assays characterizing CD4 and CD8 T cells expressing CD40L, interferon gamma (IFN-γ), interleukin 2 (IL-2), and tumor necrosis factor alpha (TNF-α). Dengue-specific T-cell responses were assessed using a method adapted from a previously described assay.^15^ PBMCs were stimulated for 2 hours by the lysate of Vero cell infected with the DENV serotype being analyzed, and medium as negative control in the presence of co-stimulatory antibodies to CD28 and CD49d. Brefeldin A was added for a subsequent 18-hour incubation to promote intracellular accumulation of cytokine. Cells were stained using fluorochrome-conjugated antibodies before enumeration by flow cytometry–specific CD4^+^ /CD8^+^ T cells expressing two or more immune markers among CD40L, IFN-γ, IL-2, and TNF-α with the background response (no stimulation) subtracted.

### Viremia and suspected/confirmed dengue.

Viremia testing was conducted at AFRIMS, Bangkok, Thailand, on blood samples collected 10 days after booster vaccination, regardless of symptoms, and at any time dengue was suspected. Quantitative nested reverse transcriptase polymerase chain reaction (RT- qPCR) was used to detect DENV RNA as previously reported.^8^,^16^ Certain blood sample containing DENV had a partial genomic sequence (*E* gene) analysis performed to characterize it as a vaccine virus or wild-type virus as previously described.^9^ The limit of detection (LOD) for the RT-qPCR assay for dengue viremia was used as the cutoff to determine positivity (assay value was ≥ LOD). Assay cutoffs were as follows: DENV-1: 2.70 log genome equivalents (GEQ)/mL, DENV-2: 2.70 log GEQ/mL, DENV-3: 2.70 log GEQ/mL, and DENV-4: 3.40 log GEQ/mL.

The case definition of laboratory-confirmed dengue included the following criteria: 1) the subject had a fever (axillary temperature ≥ 38°C) measured at least once on three successive days, 2) there was no reasonably certain alternative diagnosis by a qualified physician, and 3) DENV was detected in blood by RT-PCR or virus culture.

Because of the possibility of study subjects being infected with a wild-type DENV during these studies, dengue with onset outside of the 4- to 21-day postvaccination period was presumed to be caused by wild-type DENV. Conversely, dengue with onset from 4 to 21 days after vaccination was considered to be caused by vaccine virus. The presumptive attribution of dengue to vaccine virus could be revised if nucleotide sequence analysis of the DENV recovered in serum demonstrated a distant phylogenetic relationship to the vaccine virus of the same serotype. At other times during the follow-up for both studies, if dengue was suspected, parents were asked to contact the investigator so that a blood sample for viremia could be collected with parental consent.

### Data analysis.

This was a small, descriptive study designed to execute the sponsor's safety surveillance and gather observations on long-term safety, immunogenicity, and boosting potential of the vaccine candidates. All statistical analyses were performed using SAS software (versions 9.1 and 9.2; SAS Institute Inc., Cary, NC).

#### Safety analyses.

The safety analyses were performed on all vaccinated subjects. The overall percentages of subjects reporting a solicited adverse event (AE) 21 days after booster vaccination were tabulated with exact 95% confidence intervals (CIs), and unsolicited AEs, SAEs, and hospitalizations for suspected dengue were described. The proportion of subjects with abnormal safety laboratory results and those with viremia 30 days after vaccination were reported. We estimated the proportion of Infant study control subjects who sustained a dengue infection between years 1 and 4 by defining infection as a 4-fold increase in DENV neutralizing antibody titer for at least one serotype.

#### Immunogenicity analysis.

The immunogenicity analyses included subjects who complied with the procedures defined in the protocol and for whom assay results were available for at least one serological test after booster vaccination.

Seropositivity (titer ≥ 1:10) rates and the proportion of subjects with a tetravalent response were calculated by group, with exact 95% CIs. The proportion of subjects with a tetravalent response was defined, at each time point, as the percentage of subjects with PRNT neutralizing antibody titers ≥ 1:10 for all four DENV serotypes. Geometric mean titers (GMTs) by group, reported with 95% CIs, were computed for each time point by taking the antilog of the mean of the log-transformed titers. Antibody titers below the cutoff of the assay were given an arbitrary value of half the cutoff for the purpose of GMT calculation. The GMT and seropositivity rate for JEV neutralizing antibody titers measured at year 1 were calculated with 95% CIs. Frequencies of DENV-specific memory B cells and DENV-specific CD4^+^ and CD8^+^ T cells were calculated.

## Results

### Study population.

Refer to Figure 1 for study populations and Figure 2 for subject disposition.

### Vaccine safety and reactogenicity of the booster dose.

Refer to Table 2 for a summary of solicited injection site reactions that were reported in both studies. Most injection site reactions were mild to moderate, transient, and lasted for two or fewer days.

Refer to Table 3 for a summary of solicited general AEs that were reported in both studies. Most AEs lasted 2 days or less and were of mild to moderate intensity, and all but one were assessed as being causally related to vaccination (data not shown). Grade 3 solicited general AEs were not reported in the Child study. The following grade 3 solicited general AEs were reported in the Infant study, all in the full-dose group: fever (13.8% [*N* = 4]), headache (6.9%), arthralgia (3.4%), and nausea (3.4%) (data not shown). Among the four subjects with a grade 3 fever, two were viremic at day 10 and are described in section Vaccine-related viremia).

In the Child study, dizziness and rhinorrhea were the most frequently reported unsolicited AEs (reported by three and two of the seven children, respectively). The cases of dizziness were evaluated as causally related to vaccination, and rhinorrhea was unrelated to vaccination. None of the unsolicited AEs were of grade 3. In the Infant study, there were no grade 3 unsolicited AEs reported in the full-dose group, and 1one case of grade 3 bronchitis was reported in the 1/10 dose group (unrelated to vaccination). Mild to moderate respiratory infections and symptoms (rhinorrhea, upper respiratory tract infection, and nasopharyngitis) and diarrhea were the most frequently reported unsolicited AEs, with an occurrence of approximately 10–14% (data not shown).

In the Child study, the only dengue physical examination finding reported during the follow-up period was lymphadenopathy, which was also present at baseline (i.e., before vaccination in the primary study) and at 30 days post booster vaccination for three subjects (42.9%) and again at either year 2 or year 3. One other subject had a finding of lymphadenopathy that occurred only at year 3 (negative at baseline and all other time points). The most frequently occurring examination finding 30 days post booster vaccination period in the Infant study was localized lymphadenopathy reported in 18 subjects (62.1%) in the full-dose group and three (75.0%) in the 1/10 dose group. None of the dengue physical examination findings were clinically noteworthy. There were no findings for conjunctival hemorrhage, conjunctival injection, hepatomegaly, skin or mucosal hemorrhage, or splenomegaly in the Infant study.

### Vaccine-related viremia.

DENV RNA was detected in the serum sample of one of seven children in the Child study during the scheduled testing at day 10 post booster vaccination. This subject had DENV-4 viral RNA (4.34 log GEQ/mL of serum) of vaccine origin based on genotypic analysis. The child also had fever, fatigue, and headache. By day 11, the child had no symptoms. No abnormal or clinically significant laboratory results were observed. The child was absent from school for 1 day as a result of the symptoms. This case, not meeting the prespecified case definition for dengue (i.e., did not have a fever measured at least once on three successive days) was classified as undifferentiated fever (viral syndrome).

In the Infant study, dengue viremia was detected in three subjects (full-dose group) at the scheduled day 10 post booster vaccination testing. As per protocol for the Infant study, because these cases occurred on the scheduled day 10 visit, they are assumed to be vaccine-related viremia. One subject with DENV-4 RNA (4.23 log GEQ/mL of serum) at day 10 had fever (39.1°C) on day 8 lasting one day (treated with paracetamol) and headache on day 7 lasting one day. Lymphadenopathy was reported during the dengue physical examination at days 10, 21, and 30 postvaccination for this subject. One subject had DENV-2 RNA (3.00 log GEQ/mL of serum) with no dengue examination findings or any illness. The third subject had DENV-4 RNA (4.32 log GEQ/mL) measured on day 10 after booster vaccination with dengue examination findings. This subject was suspected to have dengue and is described in below section.

### Suspected and confirmed dengue cases.

In the Child study, there were no reports of hospitalization for suspected or confirmed dengue occurring in the period starting after completion of the primary vaccination study and throughout the long-term study at year 5 (ending 4 years after the booster dose).

In the Infant study, during the 30 days after the booster vaccination, one suspected case of dengue was reported following administration of the full dose DENV booster dose. The subject had a body temperature 37.5°C or greater on day 5–11 postvaccination with two consecutive days of ≥ 38°C at days 5 and 6 and again at days 9 and 10, accompanied by mild to moderate arthralgia, decreased activity, and nausea and vomiting. Safety laboratory measurements showed a high platelet count (520,000 cells/μL) on day 0 before booster dose was administered, normal on day 10, and high on day 30 (632,000 cells/μL). All other safety laboratory values were normal throughout the 30-day follow-up. DENV-4 viremia (4.32 log GEQ/mL of serum, due to vaccine virus strain) was detected at day 10 while clinical safety laboratory values were normal.

During the long-term follow-up periods in both studies, a single laboratory-confirmed dengue case was reported. A 4-year-old subject who received F17/Pre full-dose vaccine in the Infant Study presented at the hospital with fever (38.5°C), vomiting, loose stools, mild dehydration, and sunken eyes, approximately 2.7 years after the second dose (and before the booster dose). The admission platelet count of 118,000 cells/μL declined to 93,000 cells/μL on hospital day 3; serial hematocrits did not show hemoconcentration ≥ 20%. The subject was hospitalized for 8 days. The final diagnosis was laboratory-confirmed dengue fever caused by DENV-2 as confirmed by nested PCR and recovery of DENV-2 by mosquito inoculation, and corroborated with detection of an appropriate secondary antibody response to DENV (AFRIMS DENV IgM 83 ELISA units and IgG 252 ELISA units in a convalescent specimen). In this child, primary vaccination elicited neutralizing antibodies to DENV-2 that declined to a titer of 1:29 1 year after dose 2. As the subject was hospitalized approximately 1.7 years after this last result was obtained, the level of neutralizing antibody at the time of DENV-2 exposure is unknown. The markedly increased level of anti-DENV neutralizing antibody at the year 3 visit, several months after hospitalization and before the booster dose, was consistent with the hospitalized illness being dengue.

### Serious AEs.

In the Child study, no SAEs or withdrawals due to an AE were reported from the day after the primary study to the conclusion of the long-term follow-up study.

Although no SAEs were reported in the 30 days after booster vaccination in the Infant study, four subjects reported an SAE during the entire follow-up through year 4: two in the control group (pneumonia and urinary tract infection reported in year 3), and two in the full-dose group (dengue fever in year 2 reported before the booster dose, previously described, and umbilical hernia reported in year 2). None of these were related to vaccination, study participation, or lead to withdrawal.

### DENV neutralizing antibodies pre and post booster dose.

The PRNT_50_ responses in the per-protocol analyses for both studies are shown in Table 4 (Child study) and Table 5 and Supplemental Figures 1–4 (Infant study).

In the Child study, for each DENV serotype, GMTs were highest 30 days post booster dose (approximately 4-fold increase from year 1 pre booster titers) and dropped back to pre booster levels 1 year later. Two years after the booster dose, GMTs to each DENV serotype were slightly higher than the previous year titers.

One of the seven DENV recipients in the Child study was not included in the per protocol analysis because of a wild-type DENV-2 infection prior to the first dose of the vaccine in the primary study.^8^ This subject responded to the booster dose with high titers 30 days after the booster dose especially for DENV-2 and DENV-4. Titers to each DENV serotype for this subject decreased by year 2.

In the Infant study, among the full-dose vaccine recipients, GMTs to each DENV serotype increased from pre to 1 month post booster vaccination (approximately 3-fold increase for DENV-1 and DENV-3, 9-fold increase for DENV-2, and 4-fold increase for DENV-4). From 1 month post booster to 1 year post booster, DENV-1 GMTs decreased to approximately pre booster levels and DENV-2, -3, and -4 GMTs decreased but remained higher than pre booster levels.

### Control group (infant study).

A control group of 16 subjects was included in the Infant study. One month after the second dose of control vaccine, none of the 16 in the control group was seropositive to any DENV serotype. Between year 1 and year 4 of follow-up, four of 15 subjects in the control group (27%) sustained at least one DENV infection based on a ≥ 4-fold titer increase to one or more DENV types.

GMTs in the control group were low and similar for all DENV serotypes. In contrast to what was found in the control group, 28% of the subjects in the full-dose group had DENV antibody titers against all four serotypes just prior to the booster at the year 3 visit.

No control recipients had tetravalent neutralizing antibody at any time point (note that for one subject, DENV-2 titers were unknown [not measured], at the year 1 visit). One subject in the control group who was identified at the year 1 visit as having acquired multivalent DENV antibodies was found at year 4 to have late convalescent monotypic antibody to DENV-4 (possible DENV-4 infection).

### Cell-mediated immunity.

DENV-specific B- and T-cell responses, measured 2 years after the booster dose in the Child study were low to absent (data not shown).

In the Infant study, prior to the booster dose, the frequencies of DENV-specific memory B cells were low. Nevertheless, the booster dose induced a relatively low but clear frequency of DENV-specific memory B cells to each of the DENV serotypes (data not shown), which at 1 year post booster (year 4), decreased to the level obtained before the boost (year 3 pre booster).

**Figure 3.**
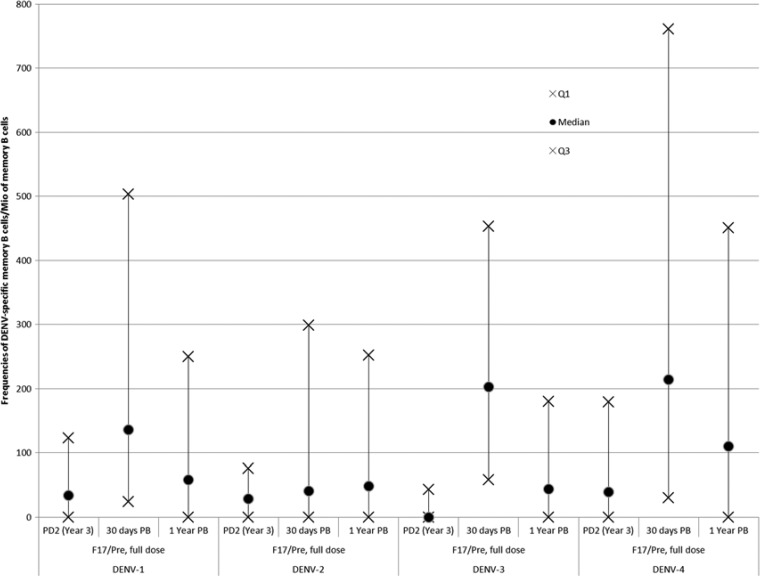
Frequencies of B-cell ELISPOT results in the Infant study (full-dose group) descriptive evaluation in terms of quartile 1 (Q1), median, and quartile 3 (Q3) of the frequencies of dengue virus (DENV) 1–4 specific memory B cells measured pre booster dose at year 3 PD2 (year 3), 30 days post booster dose (30 days PB) and 1 year post booster dose (1 year PB) in the sub-cohort of the full-dose group. Mio = million; PD2 = post-dose 2.

After the booster dose, close to 20% of subjects had a response above 1,000 DENV-specific memory B cells per million of memory B cells for each DENV serotype (versus no response pre booster). This would suggest that the initial vaccine regimen had primed a B-cell response reactive to the DENV IgE (Figure 3

).

T-cell evaluation revealed that there were no CD4^+^ or CD8^+^ T-cell responses detected after booster vaccination among the F17 recipients (data not shown).

### JEV neutralizing antibodies.

In the primary studies, subjects received two doses of JEV vaccine 1 month and 6 weeks after receiving the second dose of DENV or control vaccine. In the Child study, all subjects were seronegative to JEV at year 1, pre booster, but one-third (two of six) became seropositive for JEV antibodies later (30 days after booster vaccination with F17/Pre). At that point, all subjects received a booster dose of JEV vaccine and 12 months later, at year 2, half of the subjects (three of six) remained seropositive to JEV.

In the Infant study, approximately 11 months after the second JEV vaccine administered in the primary study, 24% of the subjects who received two doses of F17/Pre vaccine (either group) followed by two doses of JEV vaccine were seropositive to JEV, whereas 33% of the control group (two doses of JEV vaccine but no F17/Pre vaccine) were seropositive at this time point.

## Discussion

We report the first long-term safety follow-up, and booster studies of the investigational WRAIR/GSK Vaccines tetravalent DENV vaccine candidate (either original master seed [F17/Pre] or re-derived [F17]) administered to children and infants in a dengue endemic setting. We observed that a booster dose of the vaccine administered at either 1 year or more than 3 years after primary vaccination appeared safe and generally well tolerated when given to young subjects who were primed by previous F17/Pre and JEV vaccinations during childhood or infancy. Long-term safety evaluation in both studies revealed no clinically important findings and no SAEs or AE-related withdrawals following the primary and booster vaccination. Overall, AE reporting following booster vaccinations was similar to that following the primary vaccination studies.^8^,^9^

Observed clinically overt dengue illness was rare in the small sample studied; there were no cases reported in the Child study, and one breakthrough case of dengue (DENV-2) occurred in the Infant study. The single instance of dengue illness recognized was in a subject in the full-dose vaccine group whose DENV-2 neutralizing antibody titer was 1:29 1 year after the second vaccine dose, similar to the 1:28 GMT for his treatment group at the same time point. Although this breakthrough case may be an outlier, it may also suggest that the anti-DENV-2 response observed in the Infant study is not associated with durable protection.

The Infant study cohort was exposed to wild-type DENV during the study period as demonstrated by detection of 4-fold antibody increases for at least one DENV serotype in 27% of previously seronegative control group subjects (*N* = 15) from year 1 to year 4, a median surveillance duration of 53 months.

However, none of these infections in the control group resulted in hospitalization.

In each study, the proportion of subjects positive for DENV viremia measured after booster vaccination was very low and similar to that after primary vaccination (children: 0.14% post booster versus 0.43% post primary,^8^ and infants: 0.06% versus 0.14%, respectively^9^).

Although the experience is very limited, the reduction in viremia frequency suggests that antibody-mediated enhancement of infection following booster vaccination is likely to be uncommon.

In the Infant study, during the 30 days post booster vaccination, one child presented with an acute illness temporally associated with DENV-4 viremia due to the vaccine virus. There are aspects of the illness that are inconsistent with a diagnosis of clinically overt DENV infection and better explained by an illness due to another cause. The course of fever was atypical. Fever on day 0 would not plausibly be attributed to vaccine viremia. Absence of fever between days 1 and 4 may indicate response to the paracetamol (given on days 0–7); however, such a response would be unlikely if fever was due to a natural dengue infection and the same may be true for vaccine-induced dengue. Moreover, the absence of abnormal safety laboratory values and physical examination findings consistent with dengue do not support the diagnosis. The elevated platelet counts would be more indicative of an acute phase reactant to another infectious process versus dengue where white blood cell and platelet counts typically decline. Although direct correlations between quantitative levels of vaccine induced viremia and symptoms were not available, the low level of 4.32 log GEQ/mL of serum seemed unlikely to be the cause of 4 days of continued elevated body temperature, assuming the peak viremia occurred before day 10 when viremia measurement was prescheduled. Finally, the prolonged duration of illness would be inconsistent with previously observed reactogenicity after administration of the full dose F17 vaccine and point to the occurrence of an intercurrent illness.

Antibody persistence was not evident 1–3 years after primary vaccination. Neutralizing antibody responses following a primary two-dose vaccination course decreased over time in both studies. A median of 42 months after primary vaccination, just over 1/4 of the subjects receiving F17/Pre vaccine full dose had a tetravalent DENV neutralizing antibody profile as did less than 1/5 of subjects in the Child study. These data indicate that the vaccine appears to lack a suitable immunogenicity profile. The poor persistence of vaccine immunity observed in these small cohorts is similar to that observed in a larger clinical trial conducted in Puerto Rico in which children were enrolled regardless of baseline anti-DENV antibody status.^12^

Although a third dose of the dengue vaccine (either F17/Pre or F17 formulation) appears to be well tolerated, its clinical benefit remains uncertain. In the Child study, the third dose boosted DENV antibody titers that had waned over approximately 12 months since completion of the two-dose primary vaccination course; however, the boosting effect was transient. The limited response to the administration of a booster dose suggests that most subjects may have developed a sustained immune response to primary vaccination, even though not reflected in antibody persistence. Nevertheless, immunity sufficient to restrict replication of the attenuated vaccine viruses may be entirely insufficient to prevent disease upon exposure to wild-type dengue. In the Infant study, the booster dose administered after an approximate 3-year interval elicited a tetravalent response in approximately 79% of the subjects 30 days after administration, but 1 year later, only 45% of the subjects retained a tetravalent profile. Two of the four subjects who received the 1/10 dose F17/Pre vaccine for primary vaccination followed by the full dose of F17 had a tetravalent profile 30 days following booster vaccination, but this did not persist 1 year later. Similarly, in a study of adults who received a two-dose primary vaccination of the same F17 vaccine, a third dose offered 6 months later did not increase tetravalent antibody rates.^10^

Subsequent to the booster dose in the Child study, there was evidence of natural boosting between years 2 and 3, in three of seven children, presumably reflecting environmental exposure to DENV or JEV.

Many subjects in these two follow-up studies failed to maintain neutralizing antibody to four DENV serotypes at a level > 1:10, far below the 4- to 10-fold higher titers that have been estimated as offering passive protection to newborns born in a dengue-endemic region.^17^ This suggests that although memory B cells may have been generated, the response to vaccination did not stimulate the formation of long-lived plasma cells residing in the bone marrow to continuously maintain a level of potentially protective antibodies. The cell-mediated immunity data generated in this study did not allow differentiation between absence of a specific cell response from a low sensitivity assay due to ineffective antigen stimulation, and for this reason the data are inconclusive. Nevertheless, the absence of detection of a robust T-cell response and the detection of a memory B-cell response in only a minority of subjects after a booster dose is an additional factor indicating risk of vaccine failure during development.

Absence of long-lived antibody is in contrast to what is observed with other live-attenuated viral vaccines (e.g., measles vaccine), and it suggests that there may be a risk of breakthrough illness as postvaccination anti-DENV levels fall to a sub-neutralizing level, even though such illnesses were uncommon in this work or absent in a prior publication of another live dengue vaccine candidate.^18^

As noted following a two-dose JEV vaccination course administered to all subjects during the primary vaccination phase of the Infant study, the presence of JEV antibodies was more or less similar between the F17/Pre vaccine groups and the control group and the rates of JEV positivity after JEV vaccination was not dissimilar to the rates of DENV seropositivity approximately 1 year after DENV vaccination. Neutralizing antibodies were tested with JEV SA14-14-2 because it is a biosafety level 2 (BSL2) agent, and there was no access to a BSL3 facility that would be required to test for anti-Beijing strain antibodies. The use of a heterologous virus to determine neutralizing antibodies may contribute to the low titers observed. This observation is of uncertain significance, as the group sizes were small. The potential of F17 vaccine interference with JEV vaccination is unclear, however, unlikely since the JEV vaccine is inactivated. On the other hand, in the Child study, the administration of the DENV vaccine booster appeared to have elicited a modest booster response to JEV as well. Additional studies in larger cohorts will be required to evaluate the effect of prior administration of live-attenuated dengue vaccine with subsequent immunization by inactivated JEV vaccine.

## Conclusion

In these two follow-up studies of children and infants who were primed with two doses of DENV vaccine and then given a booster dose 1–3 years later, we failed to detect long-lived, multivalent anti-DENV neutralizing antibody in most subjects. Although DENV infection occurred in some control subjects as indicated by otherwise unexplained anti-DENV antibody titer increases, only a single case of dengue illness in a vaccine recipient led to hospitalization. The occurrence of DENV-2 illness in this subject who was noted to respond to vaccination with a modest level of DENV-2 neutralizing antibody may suggest that such an antibody response may not reliably predict durable protection.

The results of these two follow-up studies indicate that the live-attenuated DENV candidate vaccine jointly developed by the WRAIR and GSK did not elicit a durable primary humoral immune response. After a booster dose, neither B- or T-cell responses nor persisting multivalent anti-DENV neutralizing antibody responses were detected in most subjects.

Further development of this vaccine as a stand-alone vaccine has ended. GSK Vaccines, the WRAIR, and Bio-Manguinhos are currently developing an adjuvanted, purified, and inactivated DENV vaccine. An important step in developing this alternate vaccine approach to prevention of dengue will be to demonstrate that B- and T-cell immune responses to vaccination persist and can be boosted after an interval of several years.

## Supplementary Material

Supplemental Figures.

## Figures and Tables

**Table 1:** DENV strains, PDK passage, and viral concentration (immunofocus assay, log10 FFU/mL) of each tetravalent preparation

Virus type and PDK passage	Child study			Infant study		
	Primary F17/Pre		Booster dose F17/Pre	Primary F17/Pre		Booster dose F17
	log_10_ FFU/mL			log_10_ FFU/mL		
	Dose 1	Dose 2*	log_10_ FFU/mL	Dose 1	Dose 2†	log_10_ FFU/mL (at release)
DENV-1 (45AZ5) PDK 27	6.1	6.2	6.0	6.1 (1/10 dil 5.0)	6.2 (1/10 dil 4.9)	4.9
DENV-2 (S16803) PDK 50	6.2	6.4	6.1	6.3 (1/10 dil 5.3)	6.3 (1/10 dil 5.3)	5.3
DENV-3 (CH53489) PDK 20	5.1	5.0	5.0	4.8 (1/10 dil 4.1)	4.7 (1/10 dil 3.9)	4.7
DENV-4 (341750) PDK 6	6.3	6.1	6.1	6.0 (1/10 dil 5.0)	6.0 (1/10 dil 5.3)	5.0

DENV = dengue virus; dil = dilution; FFU = focus forming units; PDK = primary dog kidney.

Inactive ingredients for F17/Pre: 2.5% human serum albumin and 7.5% lactose as stabilizer, Eagle's Minimum Essential Medium (EMEM) cell culture medium, streptomycin, neomycin. Inactive ingredients for F17: cell culture medium EMEM, and stabilizers carbohydrates and amino acids for injection, streptomycin, neomycin and a proprietary stabilizer provided by GSK Vaccines.

* Average of two independent F17/Pre retains.

† Average of four independent F17/Pre retains (only for the undiluted preparations).

**Table 2:** Incidence of solicited injection site reactions reported during the 21 days after DENV booster administration (total vaccinated cohort)

Child study (*N* = 7)					
Injection site reaction		*n*	% (95% CIs)		
Pain	Any	3	42.9 (9.9–81.6)		
	Grade 3	0	0.0 (0.0–41.0)		
Redness	Any	3	42.9 (9.9–81.6)		
	Grade 3	0	0.0 (0.0–41.0)		
Swelling	Any	4	57.1 (18.4–90.1)		
	Grade 3	1	14.3 (0.4–57.9)		
Infant study (*N* = 33)					
		1/10 dose group (*N* = 4)		Full-dose group (*N* = 29)	
		*n*	% (95% CIs)	*n*	% (95% CIs)
Pain	Any	1	25.0 (0.6–80.6)	11	37.9 (20.7–57.7)
	Grade 3	0	0.0 (0.0–60.2)	0	0.0 (0.0–11.9)
Redness	Any	1	25.0 (0.6–80.6)	7	24.1 (10.3–43.5)
	Grade 3	0	0.0 (0.0–60.2)	0	0.0 (0.0–11.9)
Swelling	Any	1	25.0 (0.6–80.6)	9	31.0 (15.3–50.8)
	Grade 3	0	0.0 (0.0–60.2)	2	6.9 (0.8–22.8)

CIs = confidence intervals; DENV = dengue virus; *N* = number of subjects with the documented dose; n/% = number/percentage of subjects reporting any grade or grade 3 of the adverse event at least once. Any: grade 1, 2, or 3. Child study booster: F17/Pre. Infant study booster: F17 (full dose and 1/10 dose of F17/Pre given in primary study).

**Table 3:** Incidence of any grade of solicited general AEs reported during the 21 days after dengue booster vaccination (total vaccinated cohort)

Child study (*N* = 7)				
AE	*n*	% (95% CIs)		
Abdominal pain	1	14.3 (0.4–57.9)		
Arthralgia	1	14.3 (0.4–57.9)		
Fatigue	4	57.1 (18.4–90.1)		
Fever	1	14.3 (0.4–57.9)		
Headache	2	28.6 (3.7–71.0)		
Muscle aches	4	57.1 (18.4–90.1)		
Nausea	0	0.0 (0.0–41.0)		
Pain behind eyes	2	28.6 (3.7–71.0)		
Photophobia	2	28.6 (3.7–71.0)		
Pruritus	1	14.3 (0.4–57.9)		
Rash	1	14.3 (0.4–57.9)		
Vomiting	0	0.0 (0.0–41.0)		
Infant study				
	1/10 dose (*N* = 4)		Full dose (*N* = 29)	
AE	*n*	% (95% CIs)	*n*	% (95% CIs)
Abdominal pain	1	25.0 (0.6–80.6)	2	6.9 (0.8–22.8)
Arthralgia	0	0.0 (0.0–60.2)	4	13.8 (3.9–31.7)
Decreased activity	1	25.0 (0.6–80.6)	5	17.2 (5.8–35.8)
Fever	0	0.0 (0.0–60.2)	13	44.8 (26.4–64.3)
Headache	0	0.0 (0.0–60.2)	11	37.9 (20.7–57.7)
Muscle aches	0	0.0 (0.0–60.2)	7	24.1 (10.3–43.5)
Nausea	1	25.0 (0.6–80.6)	2	6.9 (0.8–22.8)
Pain behind eyes	0	0.0 (0.0–60.2)	3	10.3 (2.2–27.4)
Photophobia	0	0.0 (0.0–60.2)	1	3.4 (0.1–17.8)
Pruritus	0	0.0 (0.0–60.2)	2	6.9 (0.8–22.8)
Rash	0	0.0 (0.0–60.2)	1	3.4 (0.1–17.8)
Vomiting	1	25.0 (0.6–80.6)	5	17.2 (5.8–35.8)

AE = adverse event; CIs = confidence intervals; *N* = number of subjects with the documented dose; *n*/% = number/percentage of subjects reporting any grade of the AE at least once. Child study booster: F17/Pre. Infant study booster: F17 (full dose and 1/10 dose of F17/Pre given in primary study).

**Table 4:** Seropositivity rates and GMTs for DENV neutralizing antibodies: Child study (per protocol cohort, *N* = 6)

Timing	*n*	% Seropositive (PRNT_50_ titers ≥ 1:10) (95% CI)	GMT (95% CIs)
Antibody: DENV-1			
Pre booster year 1	1	16.7 (0.4–64.1)	6.0 (3.8–9.6)
30 days PB	5	83.3 (35.9–99.6)	28.2 (6.6–121.3)
1 year PB (study year 2)	1	16.7 (0.4–64.1)	6.8 (3.1–15.1)
2 years PB (study year 3)	4	66.7 (22.3–95.7)	18.0 (5.2–63.0)
Antibody: DENV-2			
Pre booster year 1	3	50.0 (11.8–88.2)	16.8 (3.4–82.5)
30 days PB	6	100 (54.1–100)	94.4 (24.0–370.3)
1 year PB (study year 2)	4	66.7 (22.3–95.7)	20.8 (4.6–92.9)
2 years PB (study year 3)	5	83.3 (35.9–99.6)	36.3 (7.0–186.8)
Antibody: DENV-3			
Pre booster year 1	2	33.3 (4.3–77.7)	12.8 (2.1–77.5)
30 days PB	6	100 (54.1–100)	67.2 (9.3–487.2)
1 year PB (study year 2)	2	33.3 (4.3–77.7)	9.4 (2.8–31.7)
2 years PB (study year 3)	3	50.0 (11.8–88.2)	12.9 (4.1–41.3)
Antibody: DENV-4			
Pre booster year 1	3	50.0 (11.8–88.2)	27.0 (3.6–200.7)
30 days PB	6	100 (54.1–100)	112.0 (26.5–473.1)
1 year PB (study year 2)	3	50.0 (11.8–88.2)	25.4 (3.5–182.1)
2 years PB (study year 3)	6	100 (54.1–100)	70.4 (23.9–207.4)

CIs = confidence intervals; DENV = dengue virus; GMT = geometric mean antibody titer calculated on all subjects (*N* = 6); *N* = number of subjects with available results; *n*/% = number/percentage of subjects with titer above the assay cutoff; PB = post booster; PRNT_50_ = plaque-reduction neutralization test. Pre booster year 1 = pre booster (year 1, before booster administration).

**Table 5:** Seropositivity rates and GMTs for DENV neutralizing antibodies: Infant study (per protocol cohort)

Group	Timing	*N*	*n*	% Seropositive (PRNT_50_ titer > 1:10) (95% CIs)	GMT (95% CIs)
Antibody: DENV-1					
1/10 dose	PD2 (month 7)*	4	1	25.0 (0.6–80.6)	6.1 (3.3–11.4)
	PD2 (study year 1)	4	0	0.0 (0.0–60.2)	5.0 (5.0–5.0)
	PD2 (study year 3)	4	0	0.0 (0.0–60.2)	5.0 (5.0–5.0)
	30 days PB (study year 3, days 30)	4	2	50.0 (6.8–93.2)	15.0 (1.3–179.1)
	1 year PB (study year 4)	4	1	25.0 (0.6–80.6)	7.2 (2.2–23.5)
Full dose	PD2 (month 7)*	29	16	55.2 (35.7–73.6)	20.7 (11.4–37.7)
	PD2 (study year 1)	30	7	23.3 (9.9–42.3)	7.5 (5.5–10.2)
	PD2 (study year 3)	28	12	42.9 (24.5–62.8)	12.5 (7.7–20.1)
	30 days PB (study year 3, days 30)	28	24	85.7 (67.3–96.0)	36.7 (22.7–59.2)
	1 year PB (study year 4)	29	14	48.3 (29.4–67.5)	16.7 (9.1–30.5)
Control	PD2 (month 7)*	16	0	0.0 (0.0–20.6)	5.0 (5.0–5.0)
	PD2 (study year 1)	15	1	6.7 (0.2–31.9)	5.9 (4.1–8.6)
	Study year 4	15	1	6.7 (0.2–31.9)	5.6 (4.4–7.3)
Antibody: DENV-2					
1/10 dose	PD2 (month 7)*	4	4	100 (39.8–100)	173.6 (50.1–601.5)
	PD2 (study year 1)	4	3	75.0 (19.4–99.4)	16.3 (2.3–113.3)
	PD2 (study year 3)	4	2	50.0 (6.8–93.2)	12.8 (2.2–73.7)
	30 days PB (study year 3, days 30)	4	4	100 (39.8–100)	121.3 (18.5–794.4)
	1 year PB (study year 4)	4	3	75.0 (19.4–99.4)	23.6 (3.1–183.0)
Full dose	PD2 (month 7)*	28	28	100 (87.7–100)	239.6 (163.6–351.1)
	PD2 (study year 1)	30	28	93.3 (77.9–99.2)	28.4 (20.8–38.6)
	PD2 (study year 3)	28	18	64.3 (44.1–81.4)	18.1 (11.8–27.7)
	30 days PB (study year 3, days 30)	29	29	100 (88.1–100)	162.3 (121.1–217.4)
	1 year PB (study year 4)	29	29	100 (88.1–100)	116.0 (80.2–167.8)
Control	PD2 (month 7)*	16	0	0.0 (0.0–20.6)	5.0 (5.0–5.0)
	PD2 (study year 1)	14	0	0.0 (0.0–23.2)	5.0 (5.0–5.0)
	Study year 4	15	3	20.0 (4.3–48.1)	9.4 (4.3–20.2)
Antibody: DENV-3					
1/10 dose	PD2 (month 7)*	4	0	0.0 (0.0–60.2)	5.0 (5.0–5.0)
	PD2 (study year 1)	4	0	0.0 (0.0–60.2)	5.0 (5.0–5.0)
	PD2 (study year 3)	4	1	25.0 (0.6–80.6)	6.6 (2.7–15.8)
	30 days PB (study year 3, days 30)	4	4	100 (39.8–100)	19.0 (11.7–31.1)
	1 year PB (study year 4)	4	2	50.0 (6.8–93.2)	9.1 (3.0–27.2)
Full dose	PD2 (month 7)*	29	25	86.2 (68.3–96.1)	29.2 (18.6–45.7)
	PD2 (study year 1)	30	7	23.3 (9.9–42.3)	7.8 (5.5–11.0)
	PD2 (study year 3)	28	9	32.1 (15.9–52.4)	8.9 (6.2–12.6)
	30 days PB (study year 3, days 30)	28	25	89.3 (71.8–97.7)	28.3 (18.3–44.0)
	1 year PB (study year 4)	29	20	69.0 (49.2–84.7)	19.6 (11.8–32.7)
Control	PD2 (month 7)*	16	0	0.0 (0.0–20.6)	5.0 (5.0–5.0)
	PD2 (study year 1)	15	1	6.7 (0.2–31.9)	5.5 (4.5–6.7)
	Study year 4	15	4	26.7 (7.8–55.1)	8.2 (4.6–14.7)
Antibody: DENV-4					
1/10 dose	PD2 (month 7)*	4	3	75.0 (19.4–99.4)	31.9 (2.5–408.2)
	PD2 (study year 1)	4	1	25.0 (0.6–80.6)	9.8 (1.1–84.9)
	PD2 (study year 3)	4	1	25.0 (0.6–80.6)	12.2 (0.7–210.7)
	30 days PB (study year 3, days 30)	4	3	75.0 (19.4–99.4)	27.1 (2.5–288.9)
	1 year PB (study year 4)	4	1	25.0 (0.6–80.6)	14.7 (0.5–451.5)
Full dose	PD2 (month 7)*	28	27	96.4 (81.7–99.9)	52.5 (29.0–95.0)
	PD2 (study year 1)	30	13	43.3 (25.5–62.6)	13.4 (8.1–22.4)
	PD2 (study year 3)	29	14	48.3 (29.4–67.5)	16.6 (9.4–29.3)
	30 days PB (study year 3, days 30)	29	29	100 (88.1–100)	77.0 (50.9–116.5)
	1 year PB (study year 4)	29	24	82.8 (64.2–94.2)	36.8 (23.3–58.2)
Control	PD2 (month 7)*	16	0	0.0 (0.0–20.6)	5.0 (5.0–5.0)
	PD2 (study year 1)	15	1	6.7 (0.2–31.9)	7.0 (3.4–14.4)
	Study year 4	15	2	13.3 (1.7–40.5)	6.1 (4.5–8.3)

CIs = confidence intervals; DENV = dengue virus; GMT = geometric mean antibody titer calculated on all subjects; *N* = number of subjects with available results; *n*/% = number/percentage of subjects with titer above the assay cutoff; PB = post booster; PD2 = post dose 2; PRNT_50_ = plaque-reduction neutralization test.

* PD2 (month 7) data from primary study.
