# Supplementary material for: Long-Term Safety and Immunogenicity of a Tetravalent Live-Attenuated Dengue Vaccine and Evaluation of a Booster Dose Administered to Healthy Thai Children
Source: Am J Trop Med Hyg. 2016 Jun 1;94(6):1348–58. doi: 10.4269/ajtmh.15-0659 (PMC4889756; doi:10.4269/ajtmh.15-0659)
Supplement: Supplementary file 1 [file SD7.pdf]

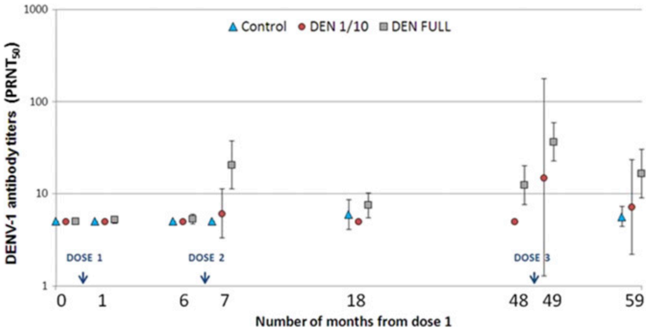

Supplemental Figure 1. Infant study: Evolution of DENV-1 neutralizing antibody GMTs with 95% CI.

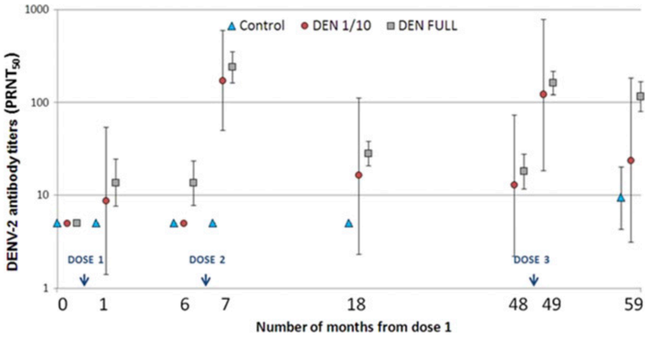

Supplemental Figure 2. Infant study: Evolution of DENV-2 neutralizing antibody GMTs with 95% CI.

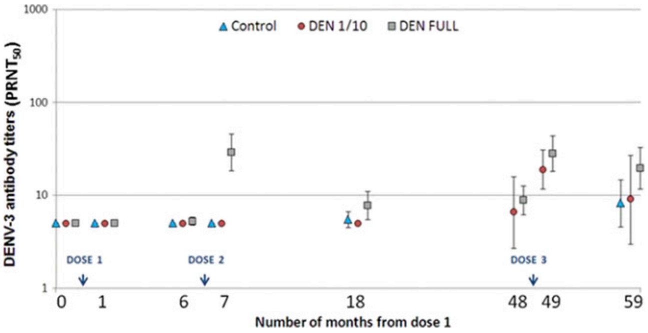

Supplemental Figure 3. Infant study: Evolution of DENV-3 neutralizing antibody GMTs with 95% CI.

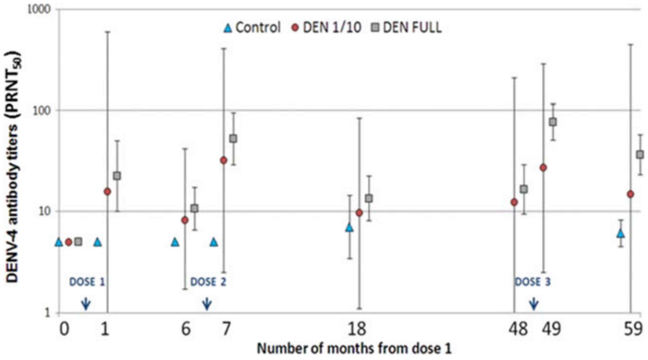

Supplemental Figure 4. Infant study: Evolution of DENV-4 neutralizing antibody GMTs with 95% CI.
